# Home-Advantage during COVID-19: An Analysis in Portuguese Football League

**DOI:** 10.3390/ijerph18073761

**Published:** 2021-04-04

**Authors:** Rui Matos, Diogo Monteiro, Raul Antunes, Diogo Mendes, João Botas, João Clemente, Nuno Amaro

**Affiliations:** 1 Life Quality Research Centre (CIEQV), IPSantarém/IPLeiria, 2040-413 Rio Maior, Portugal; raul.antunes@ipleiria.pt (R.A.); diogo.l.mendes@ipleiria.pt (D.M.); nuno.amaro@ipleiria.pt (N.A.); 2 ESECS—Polytechnic Institute of Leiria, 2411-901 Leiria, Portugal; diogo.monteiro@ipleiria.pt (D.M.); joao.s.botas@gmail.com (J.B.); joaoclemente94@hotmail.com (J.C.); 3 Research Center in Sports Sciences, Health Sciences and Human Development (CIDESD), University Institute of Maia, ISMAI, 4475-690 Maia, Portugal; 4 Center for Innovative Care and Health Technology (ciTechCare), Polytechnic of Leiria, 2410-541 Leiria, Portugal

**Keywords:** home advantage, attendance, pandemic, football, Portuguese football league

## Abstract

Covid-19 pandemic forced, at the final rounds of 2019–2020 season, in many different sport leagues worldwide, teams to play without an audience. Therefore, the present paper aims to compare the home advantage score in the last ten rounds in 2019–2020 season with the first 24 rounds in same season using Pollard’s (1986) and Matos et al.’s (2020) methods. In addition, comparisons across different seasons (2016–2017; 2017–2018; 2018–2019 and 2019–2020) using the same methods were also analyzed. Results showed no differences (*p* > 0.05) between first 24 rounds and the last 10 in 2019–2020 season as well as in the 3 previous seasons. With Pollard’s method, no differences (*p* > 0.05) were also found among those four seasons on global (all 34 rounds) home advantage. However, a significance difference between 2017–2018 and 2019–2020 (*p* < 0.05) was founded using Matos et al.’s (2020) method, which is an indicator of the importance of using complementary methods when analyzing the same realities. Overall, despite what might be expectable from recent findings, the lack of an audience in the last 10 rounds of Portuguese Football League 2019–2020 season, due to COVID-19 pandemic, did not affect home advantage. Therefore, future studies could try to analyze other different variables in Portuguese Football League, such as referees’ behaviors, rules changing (e.g., the possibility of making five substitutions, instead of three), crowd dimension and density as well as include variables about odds as forecasts in football being played without crowds.

## 1. Introduction

Home advantage in sport is a wide-spread phenomenon. Studying home advantage in football (soccer), Pollard [1] firstly introduced a measure to quantify “home advantage” by expressing the number of points won at home as a percentage of all points won at home and away. Thus, there will exist home advantage whenever teams (in general or in individual analysis), in a balanced schedule competition, commonly known as double round-robin format (meaning there is an equal number of games played at home and away from home, in a reciprocal basis amongst all the teams), win more than 50% of the total points won at home. A figure of 50% would mean no advantage (and no disadvantage, either) of playing at home and figures below 50% would mean home disadvantage. To overcome this constraint (and even contradiction) that represents the fact of reporting that, for instance, in a certain competition home advantage was 50% when, in fact, there was no advantage (and disadvantage) at all, Matos, Amaro and Pollard [2] proposed an alternative method. Its concept relies on determining home advantage according to the direct dependence of home performance upon away performance. Thus, if, e.g., in a championship teams won, at home, 60 points and away 40 points, that would mean that home advantage would be, in fact, 50%. This figure would come from the finding that, at home, teams won 50% more points than they won away from home [(60−40)/40)]. With Pollard’s [1] method, home advantage would be presented as 60% (60/100). Further in this study, we will use both methods seeking for possible different findings according to the use of one or another method.

Over the years, several different countries and professional team sports have shown that teams, globally, win more points when playing at home than away [3,4,5,6]. Different factors have been pointed out to explain home advantage: crowd support, as well as territoriality, familiarity with the stadium, travel fatigue and referees’ bias are amongst them [7]. Nevertheless, home advantage, namely in all English football leagues, has declined significantly over the last 45 years [8], probably due to augmented professionalism and decreased identification of the local community with clubs, among other factors [9]. Furthermore, Pollard et al. [5] have shown that sports played in open spaces (as football is) have lower home advantage. Nevertheless, home advantage is still present around the globe.

In many different sports leagues worldwide at the final stages of the 2019–2020 season, Covid-19 pandemic forced teams to play without an audience, football being no exception. This represented an (almost) unique opportunity to get a better insight about the effective importance of crowd support on home advantage. Before the 2019–2020 season, only sporadically were there games played without an audience, namely when one team was punished with that sanction (closed door game). Thus, it was not easy, before 2019–2020, to have a considerable amount of data that could be used to understand, more thoroughly, this influence. To the best of our knowledge, the study of van de Ven [10] was the only one undertaken before 2019–2020 on football. The author analyzed 20 games of 2006–2007 Italian Championships season (7 on Series A, 13 on Series B) performed without an audience, since the stadiums of the home team did not comply with safety rules. Maybe surprisingly (according to the more recent findings that will be reported further on), van de Ven [10] found that crowd support was not a necessary condition for the home advantage. This conclusion was achieved when comparing home advantage without an audience with home advantage with an audience where games occurred against competitors that were of similar quality to those opponents (based on final classification). Furthermore, the author analyzed Italian derbies where teams (e.g., AS Roma and Lazio Romano) shared the same stadium, from season 1988–1989 to season 2003–2004. Interestingly, home advantage was absent in these games.

Thus, this was another indicator that, apparently, more important that crowd support (in these derbies, the team that plays home has more supporters, due to tickets selling’ rights) would be the familiarity with the stadium, which was equal in these shared stadium’ derbies. This suggests that crowd support is not a necessary precondition for the home advantage. More recently, McCarrick, Bilalic, Neave, and Wolfson [11] studied all European leagues that finished 2019–2020 season without an audience. Although no individual analysis by country was available, global results revealed that, with an audience, teams won on average 0.39 points per game more at home than away, a figure that diminished to only 0.22 points more at home than away without an audience. Focusing a specific league, Tilp and Thaller [12] studied the home advantage during the Covid-19 lock-down in the German Bundesliga in 2019/20 season. Interestingly, home advantage (expressed as the number of points won home relative to the total of points won home and away), present in the previous 25 rounds with an audience (54.35%), changed to a home disadvantage (only 44.10% of the total number of points were gained home) in the last 9 rounds without assistance.

Thus, data about audience relation with home advantage requires further investigation, since results seem, as seen, somehow contradictory. The term relation was purposely used, instead of influence on. In fact, despite crowd support was previously proposed as a major factor determinant to home advantage [7], causality is unclear. For instance, crowd size may influence performance but, conversely, it is also likely that better performance brings more audience to the stadium.

Therefore, the present paper aims to compare the home advantage score in the last ten rounds in 2019–2020 season with the first 24 rounds in the same season using Pollard (1986) and Matos et al. (2020) methods. In addition, comparisons across different seasons (2016–2017; 2017–2018; 2018–2019 and 2019–2020) using the same methods was also analyzed. Specifically, it is hypothesized that: (a) due to the lack of an audience, home advantage will diminish significantly, since the presence of public, as previously stated (exception for van de Ven [10] study), is one of the main factors that literature has found to have higher influence on the existence of home advantage. Additionally, the study also aims to detect if, as pointed out before about other football leagues, home advantage in the Portuguese Football League is in a descendent trajectory throughout the last four seasons. Thus, since there is no strong reason to suppose that, in Portugal, home advantage will have a different behavior from the other professional football leagues, it is hypothesized that, as well, a descendent tendency on home advantage results over the last 4 seasons will be confirmed; (b) despite the positive (mathematic) association between Pollards’ (1986) and Matos et al. (2020) method, recently Matos et al. [2] declared that next step would be to assess the new method in one of the main football leagues in Europe (since their study was focused on handball). Therefore, it is hypothesized that some differences may arise when comparing home advantage with one and with the other method.

## 2. Methods

In 2019/20, the last 10 rounds of Portuguese Football League were played without an audience, oppositely to the first 24. Thus, we wanted to compare the Home Advantage on these two different periods. However, the last rounds of championships have decisive characteristics, contrary to initial ones. In fact, the last rounds determine which teams suffer relegation, which achieve qualification for European competitions and so on. Therefore, we searched for possible pre-existent differences on initial and final rounds home advantage prior to pandemic seasons. This would prevent us to reach mislead conclusions and would work as a baseline for 2019/20 analysis. Consequently, we searched for this behavior on the last three seasons before season 2019/20. That is why we have extended the comparison between last 10 rounds and first 24 to 3 previous seasons. In order to, somehow, attenuate the possible influence of different teams’ ability and/or quality of opposition on the home advantage effect, we have conducted an analysis on the same seasons comparing the last 10 rounds of the first half competition with last 10 of the second half. With this additional procedure, we intended to have a way of comparing the same teams having the same matches, although exchanging positions (home and away from home) between, as an additional control helping to validate home advantage determination.

### 2.1. Choice of Data Set

Final league tables for the Portuguese football League for seasons 2016/17, 2017/18, 2018/19 and 2019/2020 were accessed from the website https://www.ligaportugal.pt/pt/homepage (accessed on 29 October 2020), the official Portuguese football League site.

### 2.2. Different Methods of Quantifying Home Advantage

#### 2.2.1. Pollard’s Method (1986)

Home advantage = (H/(H + A)) ∗ 100% represents the number of points won at home expressed as a percentage of all point won at home and away. This measure is always non-negative and varies from 0% (when no points are won at home) to 100% (no points won away), with a value of 50% representing an equal number of points won at home and away (i.e., no home advantage).

#### 2.2.2. Matos, Amaro and Pollard’s Method (2020)

Home advantage = ((H − A)/A) ∗ 100%. This method [2] gives the difference between home and away points expressed as a percentage of the number of away points. It emerged because authors noticed that the obtained value with Pollard’s method does not properly relate to the advantage of playing at home, and that the reference point of no home advantage should be 0% rather than 50%. Thus, no home advantage will be represented by a value of zero, a positive value indicates the existence of a home advantage and a negative value, a home disadvantage.

### 2.3. Statistical Analysis

First, the Shapiro–Wilk test (*n* < 50) was used to analyze data distribution. Therefore, means and standard deviation were considered for all studied variables. In addition, several independent parametric tests were used to check the possible differences across variables under analysis. Specifically, independent sample t-test was performed to analyze differences in home advantage between the first 24 rounds and the last 10, as well as the last ten rounds of first half and the last ten of second half of the double round robin competition in each season under analysis. A one-way ANOVA was performed to analyze differences in home advantage across seasons. The ANOVA was complemented with the Tukey post-hoc due to all the variances due to all variances are homogeneous (Levene’s Test > 0.05). For these tests, a *p*-value less than or equal to 0.05 was considered to rejecting null hypothesis, as suggested by Ho [13]. In case of significant result, an effect size via Cohen d (independent sample t-test) and partial eta square (one-way ANOVA) will be considered [14]. Based on Cohen [15] recommendations’ the following cut-off of effect sizes were considered: trivial (0–0.19), small (0.20–0.49), medium (0.50–0.79) and large (0.80 and greater).

## 3. Results

### Preliminary Analysis

An inspection of the data revealed no missing values. In addition, the normality test through the Shapiro–Wilk test demonstrated that all variables meet normal distribution (*p* > 0.05) ensuring the condition to conduct parametric tests.

To compare home advantage before and during 2019/20 lockdown, we used two different methods to its calculation: Pollard’s method [1] and Matos et al. [2]. Table 1 shows results on home advantage on first 24 and last 10 rounds in each season under analysis. Results exhibited no differences (*p* > 0.05). However, the home advantage mean value for the last 10 rounds was higher than for the first 24, except in seasons 2016–2017 (both methods) and 2018–2019 (Matos et al. method) [2]. The Cohen d effect size was not reported since no differences were found.

Table 2 shows results on last ten rounds of first and second half of the double round-robin competition in each season. Results evidenced no differences (*p* > 0.05). Nevertheless, it is possible to verify that the home advantage mean value for the last ten rounds of the first half of the double round-robin competition was higher than for the last ten of second half, except in seasons 2017–2018 (both methods) and 2019–2020 (Matos et al.’s method). The Cohen d effect size was not reported since no differences were found.

To have a global picture of home advantage during the last four seasons, we calculated home advantage as a mean of home advantage of the different 34 rounds (Table 3). Whatever the method [1,2], we notice that there was an increase on home advantage from 2016–2017 to 2017–2018 and, after that, home advantage dropped in 2018–2019 and again in 2019–2020.

Looking for statistical differences between home advantage across the referred seasons, we can see (Table 4) that, with Pollard’s [1] method, results revealed no differences (*p* > 0.05). The partial eta square was not reported since no differences were found.

However, using Matos et al.’s [2] method, the analysis of variance revealed a significant difference in home advantage between 2017–2018 (the highest of the four seasons) and 2019–2020 (the lowest of the four seasons), with a mean difference of 105.92, *p* = 0.04 and partial eta square = 0.39. However, the effect size is small. The remaining seasons revealed no differences between them.

## 4. Discussion

The main aim of the present study was to analyze whether home advantage would be hampered by the lack of an audience at the final rounds of 2019–2020 season in Portuguese football league. The study also aimed to (i) detect if, as pointed out before about other football leagues, home advantage in Portuguese Football League was in a descendent trajectory throughout the last (4) seasons. Finally, the study also intended to (ii) search for some differences on home advantage when using Pollard’s 1986 method [1] or Matos et al.’s method [2]. Overall, the hypotheses were, respectively, that home advantage would diminish significantly; there would be a descendent tendency on home advantage results over the last 4 seasons on Portuguese Football League’ home advantage and that some differences might arise when comparing home advantage with one and with the other method.

The comparison of home advantage of the ten last rounds of 2019–2020 season, where there was no audience due to COVID-19 pandemic, with the home advantage of the first 24 rounds (with an audience), revealed no significant differences. In seasons 2016–2017, 2017–2018 and 2018–2019, although all rounds were disputed with an audience, home advantage was also similar between first 24 rounds and last 10. We wanted to see home advantage behavior in these previous seasons to see if the relation between first and last rounds, although not with the same number of rounds (24/10), would show any difference between their home advantage.

In 2019–2020, the season that was specifically under our analysis, we did not find, as said, differences. However, if those differences were present between first 24 rounds and last 10 on previous seasons, that might take us to think that the absence of an audience could have been responsible for the erasing of those differences in 2019–2020. Since that was not the case, we can speculate that not having an audience did not influence home advantage. Furthermore, the comparison between the last 10 rounds of the first half and the last 10 rounds of the second half of the double round-robin competition in each of the studied 4 seasons showed no differences. We did this comparison (10/10) since that was the opportunity to compare the same teams on the same matches, with a change on the teams that played at home or away from home. That is something that could not happen when we compared rounds with (24) and without (10) an audience.

This lack of diminishing of home advantage on rounds without an audience is contrary to McCarrick et al. [11] and Tilp and Thaller [12].

The former studied all the European Football Leagues and concluded that home advantage diminished without audience. Having studied all the leagues together may have hidden leagues’ home advantage behaviors, as is the Portuguese case. The later, focusing a specific league (the German Bundesliga, also in 2019/20 season), found results that can be considered even more dramatic. In fact, home advantage in the previous 25 rounds with an audience (54.35%), changed to a home disadvantage (a figure below 50%, concretely 44.10%) in the last 9 rounds without assistance.

However, present results follow van de Ven [10] findings, which revealed that home advantage was not affected when some Italian Leagues’ teams, back in season 2006–2007, played home without an audience by lack of compliance with safety rules.

According to Peeters and van Ours [8], in the English league the home advantage has been dropping consistently over the last 45 years. Our results show the same tendency, although our analysis has been limited to the last 4 years, with home advantage dropping from 2017–2018 until 2019–2020. Nevertheless, the global home advantage result for the Portuguese League, between seasons 1997–1998 and 2002–2003, as reported by Pollard [16], was 64.79% (Pollard’ 1986 method). In the seasons that are studied now, as can be seen on Table 1, these figures, with the same Pollard’s method, dropped from 63% in 2017–2018 to 58% in 2018–2019 and to 55% in 2019–2020.

Home advantage behavior using Matos et al. [2] method or Pollard’s [1] method is quite similar, in the sense that when home advantage gets higher (or lower) with one method, it also raises (or diminishes) with the other. Nevertheless, in general, with Matos et al.’s [2] method Home Advantage results are notably higher than with Pollard’s [1] one, something that Matos et al. [2] had already pointed out with their study on Portuguese and Spanish handball professional leagues. Using Matos et al.’s [2] method of determining home advantage, the difference between home advantage of 2017–2018 and 2019–2020 reached statistical significance. With Pollard’s method [1], no statistical differences among seasons were found.

One limitation of our study was the fact that, as previously stressed, we compared rounds and games that were necessarily different. In fact, we only had 10 rounds without an audience and not a complete season without an audience. Furthermore, and in spite of having tried to control the effect of different teams’ ability and/or quality of opposition on the home advantage effect through comparison of home advantage on last 10 rounds of first half and last 10 rounds of second half of the present competition (where the same teams played against each other’s), this effect was not effectively calculated. Other variables (e.g., possible diminishing of referee bias with absent audience pressure, technical-tactical indicators, less choice of coaches due to the illness of their athletes, higher number of allowed substitutions) should be included in future studies, especially if there is a clear decrease in home advantage, which was not the case. If 2020–2021 season is to be played entirely without an audience, that would be an excellent opportunity to see if this home advantage behavior would be the same as it seemed to be on these last 10 rounds. Nevertheless, even with these limitations, it seems that the lack of an audience in the last 10 rounds of Portuguese Football League 2019–2020 season, in general, did not affect negatively (or positively) the well-known advantage of playing at home.

All in all, the causes for the endurance of home advantage at last 10 rounds of Portuguese Football League 2019–2020 season were not analyzed. Therefore, future studies could try to analyze other different variables in Portuguese Football League, such as referees’ behaviors, rules changing (e.g., the possibility of making five substitutions, instead of three), as well as crowd dimension and density. In addition, future studies could also include variables about odds as forecasts in football being played without crowds, as recently suggested by Hegarty [17].

## 5. Conclusions

Despite what might be expectable from recent findings, the lack of an audience in the last 10 rounds of Portuguese Football League 2019–2020 season, due to COVID-19 pandemic, did not affect home advantage. Further studies are needed to understand if this tendency will remain as more and more games are being played without an audience.

Nevertheless, Portuguese Football League seems to follow the general European tendency of home advantage dropping. In 2019–2020, home advantage was at the lowest level, compared to previous 3 seasons and even more if compared with home advantage mean results between seasons 1997–1998 and 2002–2003.

The fact that with Matos et al.’s [2] determining of home advantage, the difference between home advantage of 2017–2018 and 2019–2020 reached statistical significance but not with Pollard’s method [1], where no difference between seasons revealed statistical significance, is an indicator of the importance of using complementary methods when analyzing the same realities. Although examining exclusively home advantage in Portuguese football league, the present study could lead to the following conclusions for practice: home advantage was not influenced by crowd and that reason could be particularly important for the odds as forecasts in football, athletes, coaches, and referee’s their competition preparation, since without crowd the pressure underlying competition could diminished and the balance between teams could appear. The Matos et al. [2] method to calculate home advantage can be used, since it appears to be a valid and reliable method and future studies could try and test this method both in football and other sports.

## Figures and Tables

**Table 1:** Comparison of home advantage between first 24 rounds and last 10 rounds.

Pollard’s Method (1986)					Matos et al.’ Method (2020)			
Seasons	First 24 Rounds Home Advantage M ± SD (%)	10 Last Rounds Home Advantage M ± SD (%)	t	*p*	First 24 Rounds Home Advantage M ± SD (%)	10 Last Rounds Home Advantage M ± SD (%)	t	*p*
2016–2017	59.1 ± 13.2	56.8 ± 17.3	0.43	0.67	90.20 ± 171.3	85.04 ± 190.8	0.08	0.94
2017–2018	60.2 ± 16.8	67.4 ± 19.4	−1.1	0.28	118.25 ± 196.3	210.06 ± 215.8	−1.2	0.23
2018–2019	57.6 ± 16.1	58.3 ± 13.9	−0.12	0.90	100.42 ± 231.2	73.41 ± 115.1	0.35	0.73
2019–2020	53.6 ± 15.4	58.8 ± 12.7	−0.94	0.36	27.57 ± 65.9	68.19 ± 98.2	−1.4	0.17

**Table 2:** Comparison of home advantage between last 10 rounds of first and second half of the double round-robin competition.

Pollard’s Method (1986)					Matos et al. Method (2020)			
Seasons	Last 10 Rounds 1st Half M ± SD (%)	Last 10 Rounds 2nd Half M ± SD (%)	t	*p*	Last 10 Rounds 1st Half M ± SD (%)	Last 10 Rounds 2nd Half M ± SD (%)	t	*p*
2016–2017	63.3 ± 12.5	56.8 ± 17.2	−0.52	0.61	124.40 ± 188.9	85.04 ± 190.75	0.46	0.65
2017–2018	56 ± 16.6	67.4 ± 19.4	−0.18	0.86	65.25 ± 128.3	210.66 ± 215.91	−1.8	0.08
2018–2019	59.2 ± 21.7	58.3 ± 13.9	0.16	0.88	178.48 ± 344	73.41 ± 115.14	0.32	0.37
2019–2020	60 ± 15.9	58.8 ± 12.6	−0.12	0.91	51.94 ± 70.2	68.19 ± 98.19	−0.43	0.68

**Table 3:** Home advantage in Portuguese Football League since 2016–2017 until 2019–2020.

Pollard’s Method		Matos et al.’s Method
Seasons	34 Rounds Home Advantage M ± SD (%)	34 rounds Home Advantage M ± SD (%)
2016–2017	58.44 ± 14.2	88.66 ± 174.3
2017–2018	62.29 ± 17.6	145.43 ± 203.4
2018–2019	57.79 ± 15.3	92.47 ± 202.51
2019–2020	55.15 ± 14.7	39.51 ± 77.51

**Table 4:** Differences in home advantage in Portuguese Football League across 2016–2017 until 2019–2020 seasons.

Seasons	Home Advantage Multiple Comparisons	Pollard’s Method *p*	Matos et al.’s Method *p*
2016–2017	2017–2018	0.74	0.53
	2018–2019	0.99	0.99
	2019–2020	0.82	0.64
2017–2018	2018–2019	0.63	0.59
	2019–2020	0.23	0.04
2018–2019	2019–2020	0.89	0.59

## Data Availability

Interested researchers may contact the corresponding author (rui.matos@ipleiria.pt).
